# Regulation of energy homeostasis by the ubiquitin-independent REGγ proteasome

**DOI:** 10.1038/ncomms12497

**Published:** 2016-08-11

**Authors:** Lianhui Sun, Guangjian Fan, Peipei Shan, Xiaoying Qiu, Shuxian Dong, Lujian Liao, Chunlei Yu, Tingting Wang, Xiaoyang Gu, Qian Li, Xiaoyu Song, Liu Cao, Xiaotao Li, Yongping Cui, Shengping Zhang, Chuangui Wang

**Affiliations:** 1 Institute of Translational Medicine, Shanghai General Hospital, Shanghai Jiao Tong University School of Medicine, Shanghai 201620, China; 2 Shanghai Key Laboratory of Regulatory Biology, Institute of Biomedical Sciences, East China Normal University, Shanghai 200241, China; 3 School of Life Science & Technology, China Pharmaceutical University, Nanjing 210009, China; 4 Key Laboratory of Medical Cell Biology, College of Translational Medicine, China Medical University, Shenyang 110000, China; 5 Department of Molecular and Cellular Biology, Baylor College of Medicine. One Baylor Plaza, Houston, Texas 77030, USA; 6 Key Laboratory of Cellular Physiology Ministry of Education, Shanxi Medical University, Shanxi 030001, China

## Abstract

Maintenance of energy homeostasis is essential for cell survival. Here, we report that the ATP- and ubiquitin-independent REGγ-proteasome system plays a role in maintaining energy homeostasis and cell survival during energy starvation via repressing rDNA transcription, a major intracellular energy-consuming process. Mechanistically, REGγ-proteasome limits cellular rDNA transcription and energy consumption by targeting the rDNA transcription activator SirT7 for ubiquitin-independent degradation under normal conditions. Moreover, energy starvation induces an AMPK-directed SirT7 phosphorylation and subsequent REGγ-dependent SirT7 subcellular redistribution and degradation, thereby further reducing rDNA transcription to save energy to overcome cell death. Energy starvation is a promising strategy for cancer therapy. Our report also shows that REGγ knockdown markedly improves the anti-tumour activity of energy metabolism inhibitors in mice. Our results underscore a control mechanism for an ubiquitin-independent process in maintaining energy homeostasis and cell viability under starvation conditions, suggesting that REGγ-proteasome inhibition has a potential to provide tumour-starving benefits.

## Abstract (web summary)

In conditions of energy stress cells reduce transcription of ribosomal RNA (rRNA) to maintain cell survival. Here, the authors show that energy stress induces an AMPK-dependent phosphorylation of Sirt7, which promotes its ubiquitin-independent degradation by REGγ, resulting in the down-regulation of rRNA transcription and cell survival.

## Graphical abstract

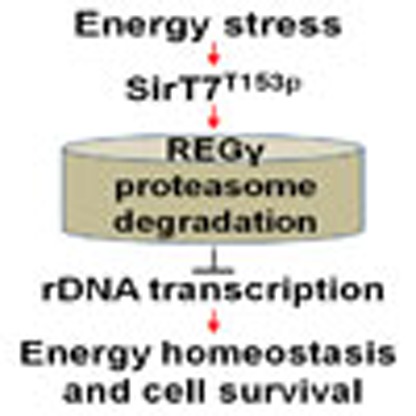

Maintenance of energy homeostasis is essential for survival and proper function of all cells. Intracellular energy homeostasis is closely related to protein degradation and synthesis. Cells mainly use the ubiquitin (Ub)-dependent proteasome system (UPS) and autophagy-lysosome system for protein degradation and the ribosomes for protein synthesis^1^. Interestingly, autophagy serves as an energy-saving process^2^, whereas both the protein synthesis and the Ub-dependent protein degradation are high energy-consuming processes^3^,^4^. Therefore, the exquisite balance between these protein degradation and synthesis systems is required to maintain proper protein and energy homeostasis. Indeed, ribosomal subunits can be targeted for degradation by both UPS^5^ and autophagy^6^. Notably, growing numbers of proteasomal substrates have been identified to be degraded by Ub-independent proteasome pathway (UIPP), and importantly, the UIPP provides cells a shortcut to degrade proteins without ATP consumption, suggesting that it serves as an energy-saving protein degradation pathway^7^. However, the functions of UIPP have not got enough attention^7^. The proteasome is a large protein complex consisting of a 20S proteolytic core and three different proteasomal activators including 19S (or PA700), 11S (or PA28, REG) and PA200. Differently, the 19S activator binds to the 20S core and mediates protein turnover in an Ub- and ATP-dependent manner, whereas the 11S proteasome mainly promotes Ub-independent protein degradation. Previous studies revealed that REGγ (or PA28γ), one of the 11S proteasomal activators^8^,^9^, promotes Ub- and ATP-independent proteasomal degradation of steroid receptor coactivator-3 and the cell cycle inhibitor p21 (refs 10, 11). Our previous study demonstrated that REGγ deficiency induces autophagy-dependent lipid degradation, indicating a role for UIPP in lipid metabolism^12^. Interestingly, starvation can increase proteasome activity with no upregulation of UPS^13^, suggesting that cell may activate UIPP to achieve energy-saving protein turnover under low energy status. However, the effectiveness of UIPP in energy homeostasis and cell fate decision under starvation remains unknown.

Limiting energy consumption in disadvantageous circumstances is critical for cell survival. Transcription of ribosomal RNA (rRNA), the first step in ribosome synthesis, is a highly energy-consuming process^14^,^15^. The TBP-TAFI complex SL1, transcription activator UBF and the RNA polymerase I (Pol I) enzyme with associated factors such as TIF1A and TIF-IC form the minimal complex required for rDNA transcription^16^,^17^,^18^,^19^.The synthesis of rRNA is tuned to match environmental nutrition conditions. Nutrients and growth factors positively regulate rRNA synthesis to adapt to cell proliferation through ERK- and mTOR-dependent TIF-IA phosphorylation^15^, whereas glucose starvation downregulates rRNA synthesis to limit energy consumption by activating AMPK-dependent phosphorylation of TIF1A^20^. Of note, during the past decade, the silent information regulator (Sir2)-like family deacetylases (also known as sirtuins) have emerged as important regulators in cell stress resistance and energy metabolism^21^,^22^,^23^,^24^. In mammals, seven sirtuins (SirT1-SirT7) have been identified. Interestingly, SirT1 forms an energy-dependent nucleolar silencing complex (eNoSC) with NML and SUV39H1 and acts as an energy-dependent repressor of rDNA transcription^4^, whereas SirT7, the only sirtuin enriched in nucleoli, associates with Pol I and UBF and positively regulates rDNA transcription^25^,^26^,^27^. Clearly, multiple signalling pathways are involved in dynamic regulation of rDNA transcription, but how these different, sometimes even antagonistic, pathways are coordinated to fine-tune rRNA synthesis to maintain energy homeostasis and cell survival under stress conditions remains to be clarified.

In this study, we reveal that REGγ-deficient cells exhibit high energy consumption and are sensitive to energy stress through increasing SirT7-directed rDNA transcription. Moreover, AMPK also plays a key role in the REGγ-SirT7 pathway in turning off rDNA transcription under energy stress conditions. Furthermore, REGγ reduction sensitizes tumours to 2DG (a competitive glycolysis inhibitor) treatment *in vivo*. Our findings disclose a role of the UIPP in maintaining cellular energy homeostasis, suggesting that REGγ is a potential therapeutic target for tumour-starving treatment.

## Results

### REGγ deficiency promotes energy consumption

**Figure 1:**
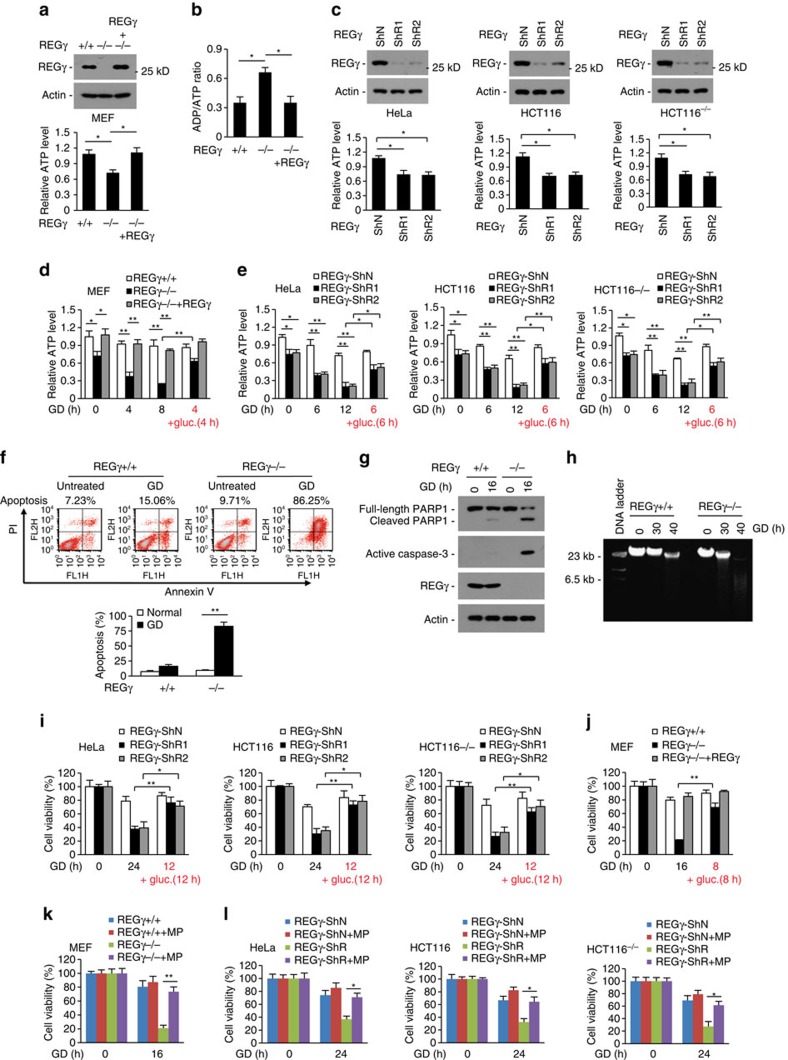
REGγ deficiency promotes energy consumption and starvation-induced cell death. (**a**–**c**) REGγ regulates cellular energy homeostasis under normal growth conditions. (A,B) MEF cells from wild-type (+/+, WT) and REGγ knockout (−/−, KO) mice were cultured in DMEM-high glucose medium. The relative intracellular ATP levels (**a**) and the cellular ADP/ATP ratios (**b**) were detected. To determine the specific effect of REGγ, REGγ-KO MEF cells were infected with lentiviral vectors expressing REGγ. Western blots show REGγ expression. (**c**) The relative intracellular ATP levels in HeLa, HCT116 and HCT116^−/−^ (p53 null) cancer cells with stable knockdown of REGγ (ShR1 or ShR2) or a vector control (ShN) cultured in DMEM-high glucose medium. Western blots show the knockdown efficiency. (**d**,**e**) REGγ deficiency promotes energy consumption. Indicated cell lines were cultured in glucose-free DMEM (glucose deprivation, GD) for the indicated time periods, or re-supplemented with glucose (+gluc.) for 4 or 6 h after GD. The relative intracellular ATP levels were analysed. (**f**-**l**) REGγ deficiency promotes energy-dependent cell death. (**f**) REGγ-WT and -KO MEF cells were treated with GD for 16 h and apoptosis was analysed by FACS. Quantitative data show percentage of apoptosis. (**g**) REGγ-WT and -KO MEF cells were treated with GD for 16 h and analysed for activated caspase-3 and poly (ADP-ribose) polymerase cleavage by western blotting. (**h**) REGγ-WT and -KO MEF cells were treated with GD for the indicated times and the large-scale DNA fragmentation was determined by agarose gel electrophoresis. (**i**,**j**) Indicated cell lines were treated with GD, or re-supplemented with glucose (+ gluc.) for the indicated time periods after GD. Cell viability was determined using MTT assay. (**k**,**l**) Indicated cells were treated with GD in the presence or absence of methyl pyruvate (MP, 10 μM) for the indicated time periods, and cell viability was analysed using MTT assay. All experiments were repeated three times, data represent mean±s.d., *P<0.05, **P<0.01; Student's *t*-test is used throughout. See also Supplementary Fig. 1.

Although the UIPP provides cells an energy-saving protein turnover shortcut, the contribution of this process in energy balance is unknown. Previous studies reported that the REGγ knockout (KO) mice displayed reduced body weight and retarded growth^28^,^29^. Our recent study showed that REGγ-KO mice exhibited over-consumption of food^12^. These observations prompted us to test the role of REGγ-proteasome in energy metabolism. Interestingly, we observed that REGγ knockout (KO) led to a significant downregulation of intracellular ATP level accompanied by an upregulation of ADP-to-ATP ratio in MEF cells, and REGγ recomplementation reversed these changes (Fig. 1a,b). Decreased level of ATP was also observed in REGγ stable knockdown human cancer cell lines (Fig. 1c). To further assess the role of REGγ in energy homeostasis, we treated cells with glucose deprivation (GD). Results showed that REGγ-KO led to a rapid and severe decrease of intracellular ATP level under GD, REGγ reconstitution significantly retarded the reduction rate of intracellular ATP level in GD-treated REGγ-KO cells, and cellular ATP level was significantly restored through glucose resupplementation in GD-treated REGγ-KO cells (Fig. 1d). Similar results were also obtained in cancer cell lines with stable REGγ knockdown (Fig. 1e). The above data indicate that REGγ plays an essential role in maintaining intracellular energy homeostasis.

### REGγ deficiency sensitizes cells to energy starvation

Intracellular energy homeostasis is crucial for cell survival. Given partial ATP depletion induces apoptosis whereas severe ATP depletion causes necrosis^30^, we analysed cell viability of REGγ-deficient cells under starvation. We observed that upon glucose deprivation REGγ-KO led to a significant increase in the percentage of late apoptotic cells (Annexin-V/PI positive) accompanied with strong upregulation of caspase-3 activation and PARP cleavage (Fig. 1f,g), and prolonged glucose starvation treatment caused severe large DNA fragmentation in REGγ-KO cells (Fig. 1h), indicating that REGγ depletion induces apoptosis and necrosis in response to energy stress.

Previous studies revealed that depletion of REGγ increases apoptosis via activation of p53 (refs 31, 32). However, we observed that REGγ knockdown facilitates GD-induced cell death in cancer cells with or without p53 (Fig. 1i), indicating that p53 is not a determinant for starvation-induced cell death in REGγ-deficient cells. In contrast, we observed that glucose resupplementation in starving cells resulted in restoring of cell survival in REGγ-knockdown and -KO cells (Fig. 1i,j), and GD-induced cell death was reversed to wild-type (WT) range by re-expressing REGγ in the KO MEF cells (Fig. 1j). Autophagy is a self-degradation process that can mediate cell death as well as survival. Our previous study found that REGγ deficiency induces autophagy^12^. However, treatment of autophagy inhibitor 3-methyladenine (3MA) had no obvious effect on REGγ KO-induced cell death under GD (Supplementary Fig. 1A). In addition, REGγ deficiency had no effect on the sensitivity of cells to glutamine or serum starvation (Supplementary Fig. 1B,C). On the other hand, treating cells with pyruvate markedly decreased GD-induced cell death in both REGγ-KO MEFs and REGγ-knockdown cell lines (Fig. 1k,l). Pyruvate not only provides energy but also serves as a peroxide scavenger^33^,^34^. However, we observed that pyruvate but not APDC (antioxidant reagent) treatment significantly increased the viability of GD-treated REGγ-knockdown cells (Supplementary Fig. 1D), suggesting that the ROS-scavenging activity of pyruvate does not play a major role in the suppression of GD-induced cell death of REGγ-deficient cells. Taken together, these results suggest that REGγ deficiency sensitizes cells to glucose starvation via disturbing intracellular energy balance.

### REGγ limits rDNA transcription

**Figure 2:**
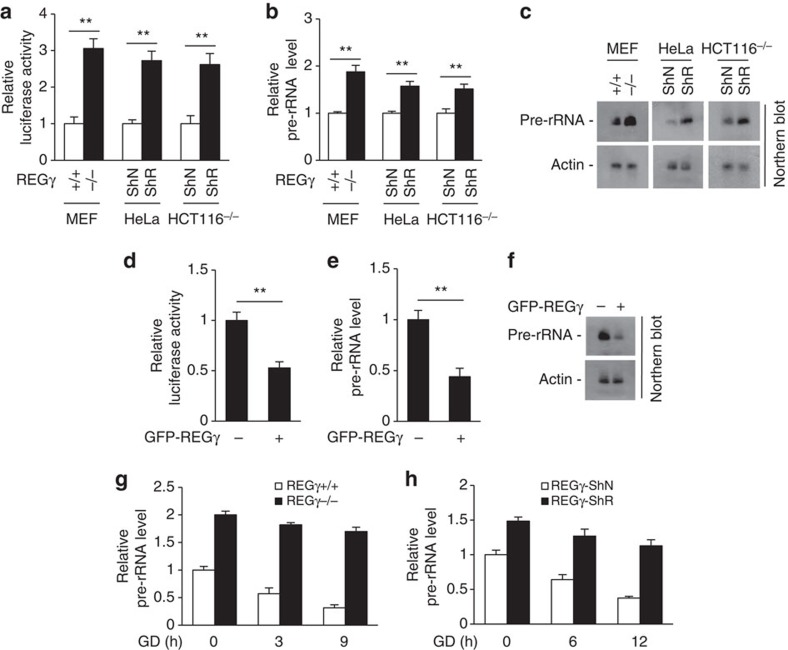
REGγ limits rDNA transcription. (**a**–**c**) REGγ deficiency enhances rDNA transcription. (**a**) Indicated stable cell lines were transfected with the rDNA-promoter luciferase reporter plasmid for 24 h and the relative luciferase activity levels were determined. (**b**,**c**) Relative pre-rRNA (47S/45S) expression levels in indicated cell lines were analysed by qRT-PCR (**b**) and northern blot (**c**). (**d**–**f**) REGγ overexpression decreases rDNA transcription. (**d**) HeLa cells were co-transfected with rDNA-promoter luciferase reporter and GFP-tagged REGγ plasmids for 24 h. The relative luciferase activity was analysed. (**e**,**f**) Levels of pre-rRNA in HeLa cells with or without GFP-REGγ overexpression were analysed by qRT-PCR (**e**) and northern blot (**f**). (**g**,**h**) REGγ deficiency delays starvation-induced pre-rRNA reduction. Indicated cell lines were treated with GD for the indicated time periods. The relative pre-rRNA transcript levels were analysed by qRT-PCR. All experiments were repeated three times; data represent mean±s.d. *P<0.05, **P<0.01, Student's *t*-test.

Downregulation of rRNA synthesis is a key process for maintaining intracellular energy homeostasis and cell survival under low energy status^4^. Next, we tested whether REGγ regulates energy homeostasis via regulating rDNA transcription. Results showed that both rDNA promoter luciferase activity (Fig. 2a) and pre-rRNA levels (Fig. 2b,c) were increased in REGγ-KO and -knockdown cells. Consistently, overexpression of REGγ decreased rDNA promoter activity (Fig. 2d) and pre-rRNA levels (Fig. 2e,f). Moreover, both REGγ-KO and -knockdown markedly blocked GD-induced reduction of pre-rRNA level (Fig. 2g,h). These results indicate that REGγ limits rDNA transcription under both normal and starvation conditions. Together with the fact that REGγ deficiency increases energy expenditure and cell death under starvation, we suggest that REGγ benefits energy saving via limiting rDNA transcription, and as a result contributes to cell survival under energy stress conditions.

### REGγ regulates SirT7 distribution and degradation

**Figure 3:**
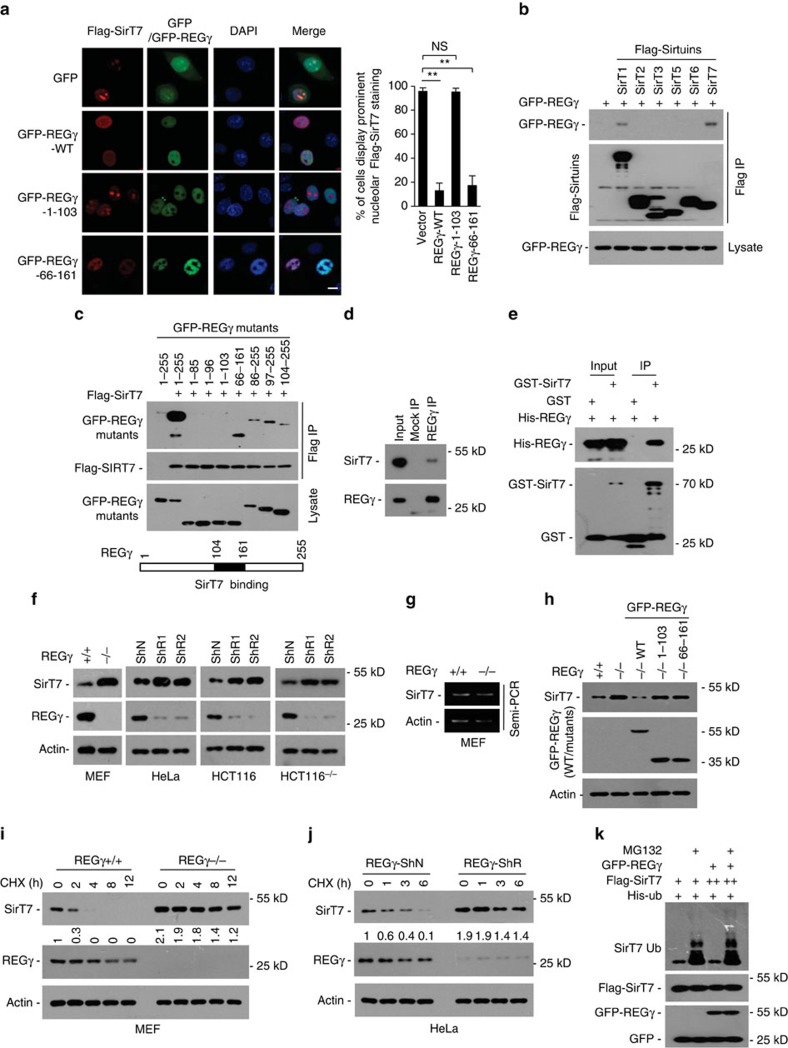
REGγ regulates SirT7 subcellular distribution and degradation. (**a**) REGγ overexpression causes SirT7 redistribution. Flag-SirT7 and GFP-REGγ (wild type, aa1-103, or aa66-161) plasmids were cotransfected to HeLa cells, and Flag-SirT7 was immunostained with anti-Flag antibody (red) and visualized by fluorescence microscopy (scale bar, 10 μm). GFP-REGγ was detected by the intrinsic GFP fluorescence. Nuclei were stained with DAPI. Graph shows the percentage of cells displaying prominent nucleolar SirT7 staining. Data represent mean±s.d., *n*=3, >100 cells were counted per replicate. NS=not significant, **P<0.01, Student's *t*-test. (**b**–**d**) REGγ interacts with SirT7. (**b**) Indicated Flag-Sirtuins and GFP-REGγ were cotransfected into 293 T cells followed by immunoprecipitation using FLAG-M2 beads and western blot using anti-GFP antibody. (**c**) REGγ-SirT7 interaction domain in REGγ was determined by cotransfection of indicated GFP-REGγ deletion mutants with Flag-SirT7 into 293 T cells followed by immunoprecipitation using FLAG-M2 beads and western blot using anti-GFP antibody. The schematic diagram indicates the region of REGγ required for SirT7 interaction. (**d**) Endogenous REGγ in HeLa cells was precipitated using anti-REGγ antibody or with IgG (Mock IP), and coprecipitated SirT7 was detected by western blot. (**e**) REGγ interacts with SirT7 *in vitro*. Recombinant His-tagged REGγ was incubated with GST-SirT7 or GST proteins at 4 °C for 4 h followed by GST pull-down and western blot. (**f**–**k**) REGγ destabilizes SirT7. (**f**) Western blot analysis of SirT7 expression in the indicated cell lines. (**g**) Semiquantitative RT-PCR analysis of relative SirT7 mRNA in REGγ-WT and -KO MEF cells. (**h**) REGγ-KO MEF cells were infected with lentivirus expressing GFP-REGγ-WT, -1-103 and -66-161 for 48 h, and endogenous SirT7 protein was detected by western blot. (**i**,**j**) Western blot analysis of lysates of indicated cell lines treated with translation inhibitor cycloheximide (CHX, 50 μg ml^−1^). Relative SirT7 band intensities were quantified through densitometry and presented. (**k**) 293 T cells transfected with His-ubiquitin, Flag-SirT7 and GFP-REGγ plasmids were treated with or without MG132 (25 μM, 4 h). Ubiquitinated proteins were precipitated using Ni-NTA beads. SirT7 ubiquitination was detected by western blot using anti-Flag antibody. To ensure equal expression of SirT7, a higher amount of SirT7 plasmid DNA (++) was cotransfected with REGγ. See also Supplementary Fig. 2.

Previous studies identified that SirT1 represses whereas SirT7 activates rDNA transcription^4^,^25^,^26^. Our recent study showed that REGγ deficiency resulted in SirT1 accumulation^12^. The fact that REGγ deficiency led to rDNA transcription activation motivated us to test whether REGγ limits rDNA transcription by negatively regulating SirT7. SirT7 is localized primarily in the nucleolus, where it interacts with Pol I and upstream control elements including UBF and B-WICH complex to improve rDNA transcription^25^,^27^. Interestingly, we observed that overexpression of both full length (aa 1-255) and central region (aa 66–161), but not the N-terminal region (aa 1-103) of REGγ, caused a marked redistribution of SirT7 into the nucleoplasm (Fig. 3a). In contrast, REGγ overexpression does not affect the subcellular localization of the nucleolar protein UBF (Supplementary Fig. 2A). Moreover, REGγ-SirT7 binding was detected in 293T cells transiently transfected with GFP-REGγ and Flag-tagged Sirtuin plasmids (Fig. 3b). Mapping the REGγ-SirT7 interaction domain using a set of REGγ deletion mutants revealed that the central region (aa 104-161) of REGγ is required for SirT7 binding (Fig. 3c). An endogenous REGγ-SirT7 complex was detectable in HeLa cells (Fig. 3d). The direct REGγ-SirT7 association was verified *in vitro* (Fig. 3e). In addition, other rDNA transcription complex proteins including UBF and MYBBP1A showed no association with REGγ (Supplementary Fig. 2B). These results indicate that REGγ specifically associates with SirT7 and regulates its subcellular distribution.

Next, we examined whether REGγ regulates SirT7 degradation. Results showed that both REGγ-KO and -knockdown resulted in elevated SirT7 expression (Fig. 3f) without altering its transcription (Fig. 3g). Moreover, restoration of full-length REGγ but not REGγ-1-103 or -66-161 (unable to activate the proteasome) repressed REGγ-KO-induced elevation in SirT7 expression (Fig. 3h). Furthermore, SirT7 protein degradation was markedly delayed in REGγ-deficient cells (Fig. 3i,j), indicating that the endogenous REGγ expression destabilizes SirT7. In addition, concomitant expression of REGγ had no effect on SirT7 ubiquitination (Fig. 3k). These data indicate that REGγ induces SirT7 degradation in an Ub-independent manner.

### REGγ limits rRNA expression and ATP consumption via SirT7

**Figure 4:**
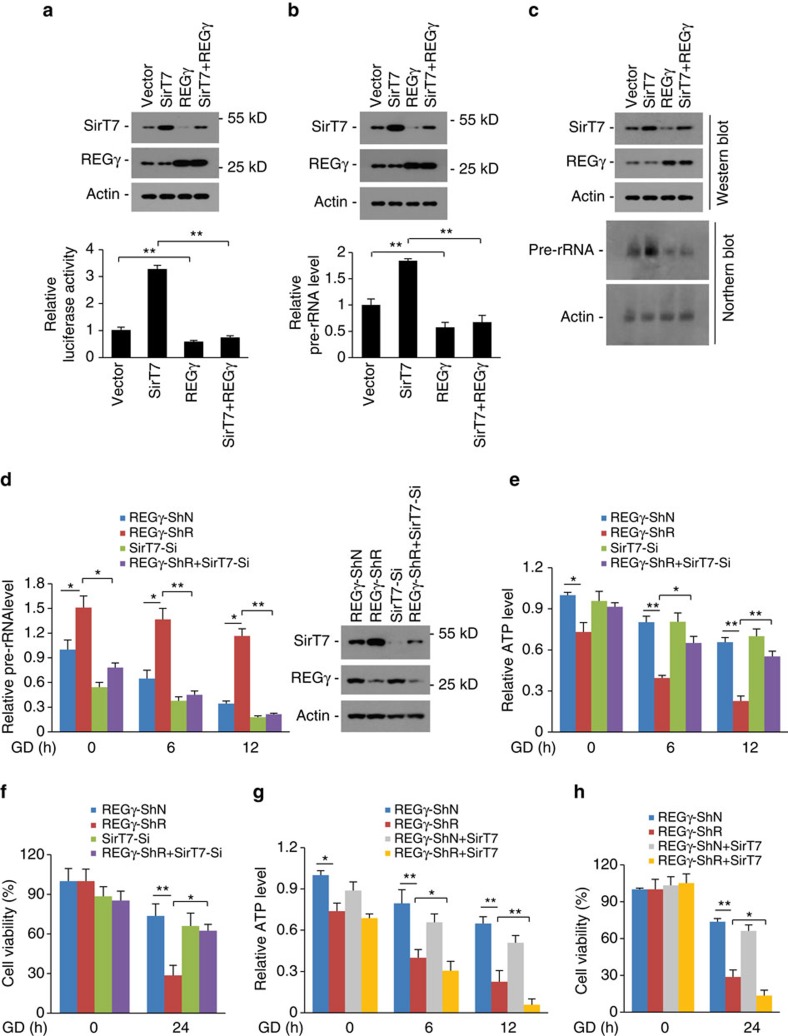
REGγ regulates rDNA transcription, energy consumption and cell death via SirT7. (**a**–**c**) REGγ inhibits SirT7 activity in rDNA transcription. (**a**) rDNA-promoter luciferase reporter plasmid was cotransfected with SirT7 and REGγ plasmids into HeLa cells for 24 h and relative luciferase activity was analysed. Representative western blots show the expression levels of SirT7 and REGγ. (**b**,**c**) HeLa cells were transfected with SirT7 and REGγ plasmids as indicated, and levels of pre-rRNA were analysed by qRT-PCR (**b**) and northern blot (**c**). Representative western blots show the expression levels of SirT7 and REGγ (upper panels). (**d**) REGγ deficiency attenuates starvation-induced reduction of pre-rRNA via SirT7 induction. REGγ-ShN and-ShR HCT116^−/−^ cells infected with or without SirT7 small hairpin RNA (SirT7-Si) expression lentivirus were treated with glucose deprivation (GD) for 6–12 h. The relative pre-rRNA levels were analysed by qRT-PCR. SirT7 knockdown efficiency was detected by western blot (right panel). (**e**,**f**) REGγ deficiency promotes starvation-induced energy-consumption and cell death via SirT7. Cells in **d** were starved for glucose for indicated time periods. The ATP consumption (**e**) and cell viability (**f**) were analysed. (**g**,**h**) SirT7 overexpression enhances starvation-induced energy consumption and cell death in REGγ-knockdown cells. Stable REGγ-ShN and -ShR HCT116^−/−^ cell lines were infected with lentivirus expressing SirT7 and cultured for 20 h, and then treated with or without GD for the indicated time periods. The relative intracellular ATP (**g**) and cell viability (**h**) were analysed. All experiments were repeated three times; data represent mean±s.d. *P<0.05, **P<0.01, Student's *t*-test.

The above data suggest that REGγ may regulate rDNA transcription via SirT7. Indeed, we observed that concomitant expression of REGγ significantly inhibited SirT7-induced upregulation of rDNA promoter activity (Fig. 4a) and pre-rRNA level (Fig. 4b,c). Under normal culture conditions, SirT7 knockdown caused a marked reduction of pre-RNA expression, and REGγ knockdown-induced upregulation of pre-rRNA expression was abolished by SirT7 double knockdown (Fig. 4d, untreated group). Furthermore, REGγ knockdown markedly retarded GD-induced pre-rRNA reduction, but the effect of REGγ-knockdown in resisting GD-induced pre-rRNA reduction was abolished after double knockdown of SirT7 (Fig. 4d). These results indicate that REGγ negatively regulates rDNA transcription through a SirT7-dependent mechanism.

Next, we examined the contribution of SirT7 in energy consumption and cell survival in REGγ-deficient cells under starvation. Results showed that double knockdown of SirT7 markedly inhibited GD-induced ATP consumption and cell death in REGγ-knockdown cells (Fig. 4e,f). Notably, SirT7 deficiency has been previously shown to induce cell death^25^,^35^,^36^. However, although transient knockdown of SirT7 caused mild induction of cell death under normal culture conditions, SirT7 knockdown showed no further effect on increasing in GD-induced cell death (Fig. 4f). In contrast, ectopic SirT7 expression markedly increased GD-induced energy consumption and cell death in REGγ-knockdown cells, but such effects were much weaker in REGγ-normal cells (Fig. 4g,h). Together, these results demonstrate that SirT7 induction in REGγ-deficient cells accelerates energy consumption and cell death under starvation.

### REGγ negatively regulates SirT7 under starvation

**Figure 5:**
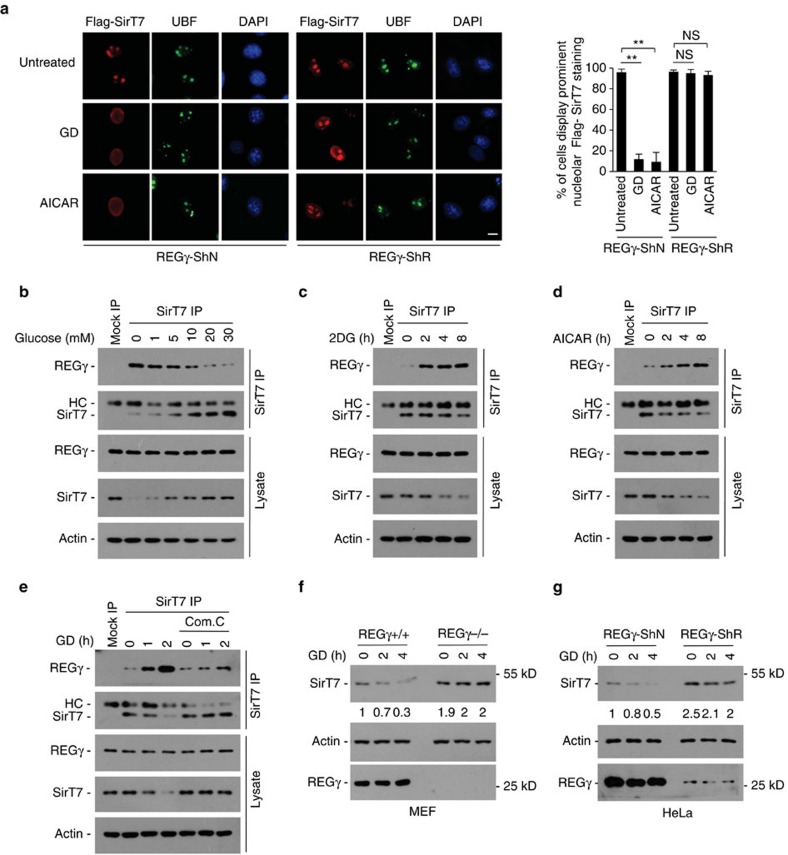
REGγ causes SirT7 nuleoplasmic redistribution and degradation under starvation. (**a**) REGγ is required for starvation-induced SirT7 nuleoplasmic redistribution. Flag-SirT7 transfected REGγ-ShN and -ShR HeLa cells were treated with glucose deprivation (GD, 12 h) or AICAR (0.5 mM, 12 h), and immunostained with anti-Flag (red) and anti-UBF (green) antibodies and visualized by fluorescence microscopy (scale bar, 10 μm). Nuclei were stained with DAPI. Graph shows the percentage of cells displaying prominent nucleolar SirT7 staining. Data represent mean±s.d., *n*=3, and at least 100 cells were counted per replicate. NS=not significant, **P<0.01, Student's *t*-test. (**b**,**c**) Energy stress increases REGγ-SirT7 association. 293 T cells were cultured in DMEM medium with different glucose concentrations (0–30 mM) for 4 h (**b**), or treated with glycolytic inhibitor 2DG in high-glucose DMEM medium for indicated times (**c**), followed by immunoprecipitation using anti-SirT7 antibody and western blot with anti-REGγ antibody. (**d**,**e**) AMPK is required for starvation-induced REGγ-SirT7 association. 293 T cells were treated with AMPK activator AICAR (0.5 mM) for indicated time periods (**d**), or with AMPK inhibitor Compound C (10 μM, 1 h) followed by glucose deprivation (GD, 1-2 h) (**e**). Cell lysates were immunoprecipitated with anti-SirT7 antibody followed by western blotting using anti-REGγ antibody. (**f**,**g**) Glucose starvation induces REGγ-dependent SirT7 degradation. Indicated cell lines were treated with GD for the indicated time periods. SirT7 degradation was analysed by western blot. See also Supplementary Fig. 3.

A recent report showed that the deacetylation of PAF53 by SirT7 activates RNA polymerase I transcription and the lack of nascent rRNA causes the release of SirT7 into the nucleoplasm under starvation^37^. The fact that REGγ overexpression caused a marked nucleolar delocalization of SirT7 promoted us to test whether REGγ regulates SirT7 redistribution under starvation. Consistent with previous work, both glucose starvation and AICAR (an AMPK activator) induced delocalization of nucleolar SirT7 to the nucleoplasm^37^. Strikingly, we observed that knockdown of REGγ markedly blocked both GD- and AICAR-induced SirT7 redistribution to the nucleoplasm, whereas UBF showed no changes of its subcellular localization upon these treatments (Fig. 5a). Moreover, treatment with glucose starvation (0-10 mM), 2DG or AICAR markedly increased REGγ-SirT7 binding (Fig. 5b-d). AMPK inhibitor Compound C treatment blocked the GD-induced increase of REGγ-SirT7 binding (Fig. 5e). Furthermore, REGγ deficiency markedly reduced GD-induced SirT7 degradation (Fig. 5f,g). These results suggest that REGγ is required for GD-induced SirT7 redistribution and degradation, and AMPK activation contributes to GD-induced upregulation of REGγ-SirT7 association.

To further address whether REGγ associates with SirT7 and promotes its degradation in the nucleolus or the nucleoplasm, we treated Flag-SirT7 overexpressing HeLa cells with actinomycin D (ActD) to disrupt nucleolus structure. Results showed that ActD treatment led to SirT7 nucleoplasmic redistribution (Supplementary Fig. 3A), while REGγ deficiency abolished ActD-induced SirT7 degradation (Supplementary Fig. 3B,C). Treatment with ActD also increased REGγ-SirT7 binding (Supplementary Fig. 3D). These results demonstrate that REGγ promotes nucleoplasmic SirT7 degradation under stress conditions.

### REGγ negatively regulates SirT7 in an AMPK-dependent manner

**Figure 6:**
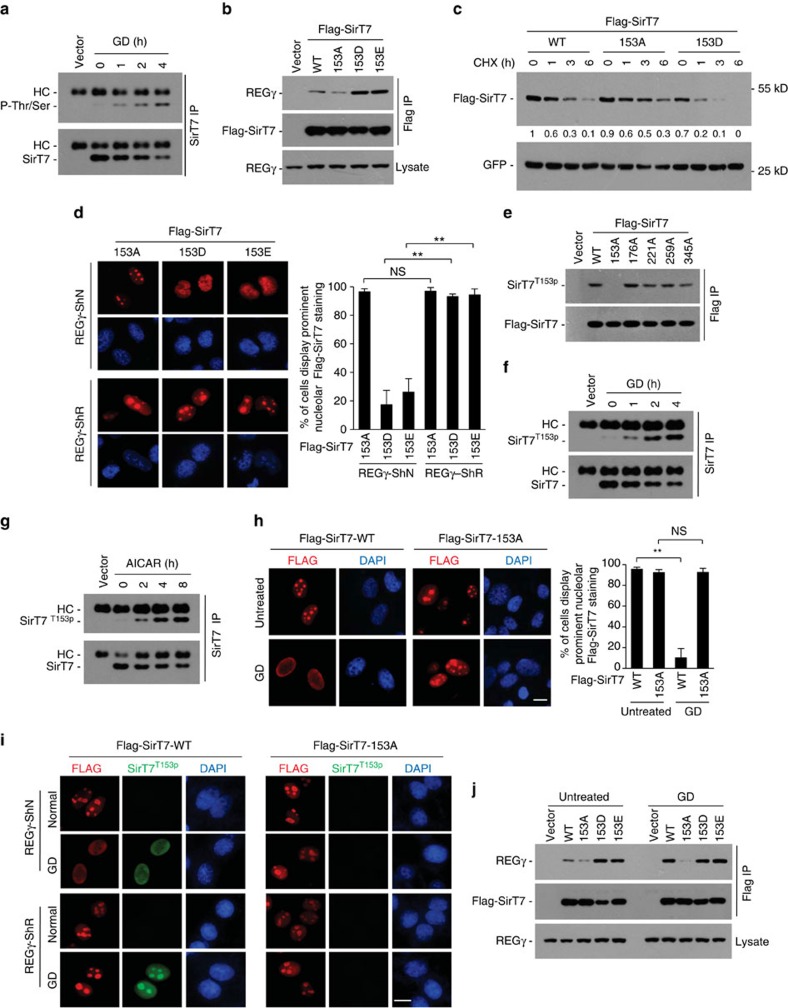
REGγ regulates SirT7 under starvation in a phosphorylation-dependent manner. (**a**) Cell lysates from GD (1–4 h)-treated 293 T cells were immunoprecipitated with SirT7 followed by western blotting using anti-phospho-Ser/Thr antibody (P-Thr/Ser). (**b**) 293 T cells transfected with indicated Flag-SirT7 T153 mutants were immunoprecipitated with FLAG-M2 beads followed by REGγ western blotting. (**c**) 293 T cells transfected with indicated Flag-SirT7 plasmids were treated with cycloheximide (CHX, 50 μg ml^−1^) and analysed for SirT7 stability by anti-FLAG western blot. Relative SirT7 band intensities were quantified through densitometry and presented. (**d**) REGγ-ShN and -ShR HeLa cells transfected with indicated Flag-SirT7 mutants were immunostained with anti-Flag antibody (red) and visualized by microscopy (scale bar, 10 μm). Graph shows the percentage of cells displaying prominent nucleolar SirT7 staining. Data represent mean±s.d., *n*=3, >100 cells were counted per replicate. NS=not significant, **P<0.01, Students *t*-test. (**e**) Characterization of SirT7 T153 phosphorylation antibody (SirT7 ^T153p^). Cell lysates from 293 T cells transfected with wild-type or mutant forms of Flag-SirT7 were immunoprecipated with FLAG-M2 beads followed by western blot using antibody against SirT7 ^T153p^ or Flag. (**f**,**g**) Cell lysates from GD (**f**) or AICAR (0.5 mM) (**g**) treated 293 T cells were immunoprecipitated with anti-SirT7 antibody followed by immunoblot with anti-SirT7 ^T153p^ or anti-SirT7 antibodies. HC, heavy chain. (**h**) Flag-SirT7-WT or -T153A mutant transfected HeLa cells were treated with or without GD (12 h) followed by immunostaining with anti-Flag antibody (red). Scale bar, 10 μm. Graph shows the percentage of cells displaying prominent nucleolar SirT7 staining. Data represent mean±s.d., *n*=3, >100 cells were counted per replicate. NS=not significant, **P<0.01, Students *t*-test. (**i**) Stable REGγ-ShN and REGγ-ShR HeLa cells transfected with Flag-SirT7-WT or -153A mutant were treated with or without GD (12 h), then immunostained with anti-FLAG (red) and anti-SirT7 ^T153p^ (green) antibodies and visualized by microscopy (scale bar, 10 μm). Nuclei were stained with DAPI. The SirT7-T153A mutant was used to evaluate the specificity of the SirT7 ^T153p^ antibody. (**j**) 293T cells transfected with indicated Flag-SirT7 plasmids were treated with or without GD (1 h). Cell lysates were immunoprecipitated with FLAG-M2 beads and probed with anti-REGγ or anti-Flag antibodies. See also Supplementary Fig. 4.

Next, we tested whether phosphorylation is involved in starvation-induced SirT7-REGγ association. Results showed that glucose deprivation markedly increased the phosphorylation levels of SirT7 (Fig. 6a). We performed mass spectrometric phosphopeptide analysis of purified Flag-SirT7 transiently expressed in 293 T cells and identified S54, S166 and T284 within SirT7 as potential phosphorylation sites (Supplementary Fig. 4A,B). A previous study reported that the amino acids 344–348 (ATPLR) of SirT7 possesses a CDK1 phosphorylation site and the phosphorylation/dephosphorylation status of this region is involved in cell cycle-dependent regulation of SirT7 activity in rDNA transcription^26^. However, we observed that the phosphorylation-defective mutants (S/T to A) of these putative phosphorylation sites (S54, S166, T284, or T345) in SirT7 showed no changes in its binding affinity for REGγ (Supplementary Fig. 4C). Given that it is possible that phosphorylation sites can be missed when performing mass spectrometric phosphopeptide analysis, we generated S/T to A mutant for most of the S/T residues in SirT7. Interestingly, we observed that the SirT7-T153A mutation decreased whereas the T153D/E phosphomimetic mutations increased their binding affinity with REGγ (Fig. 6b and Supplementary Fig. 4D). Mimic phosphorylation at T153 greatly increased SirT7 degradation rate (Fig. 6c). In addition, transiently expressed SirT7-T153D/E mutants distributed throughout the nucleoplasm in REGγ-normal cells but localized mainly in the nucleolus in REGγ-knockdown cells (Fig. 6d). These results strongly suggest that the phosphorylation status of SirT7 at T153 plays a crucial role in determining its subcellular distribution, degradation and binding to REGγ.

Notably, T153 is a highly conserved amino acid residue of SirT7 in mammals (Supplementary Fig. 4E), and the amino acid sequence around Thr153 of SirT7 (THMSIT^153^) diverges from the AMPK phosphorylation consensus motif (LXRXXpS/pT). To further confirm SirT7 T153 phosphorylation under starvation, we generated a SirT7 phospho-T153 specific antibody (Fig. 6e), and found that both GD and AICAR treatment induced strong SirT7 phosphorylation at T153 (Fig. 6f,g). Moreover, elimination of SirT7 phosphorylation at T153 (T153A) markedly blocked GD-induced SirT7 nucleoplasmic redistribution (Fig. 6h). Immunostaining of SirT7 T153 phosphorylation revealed that T153 phosphorylation of SirT7 was nearly undetectable under normal growth condition, and the T153-phosphorylated SirT7 induced by GD predominantly localized to the nucleoplasm in the REGγ-normal cells but still mainly accumulated in the nucleoli in the REGγ-knockdown cells (Fig. 6i). Furthermore, the SirT7-T153A mutant, but not the T153D/E mutants, markedly attenuated its binding to REGγ under GD treatment (Fig. 6j). In addition, SirT7 knockdown and recomplementation analyses showed that restoring SirT7-153A mutant in SirT7 knockdown cells led to a statistically significant inhibition of GD-induced repression of rDNA transcription when compared to cells recomplemented with WT (Supplementary Fig. 4F). Taken together, these results revealed that SirT7 phosphorylation at T153 causes a REGγ-dependent nucleoplasmic retaining and degradation of SirT7, and thereby contributes to turning off rDNA transcription under starvation.

**Figure 7:**
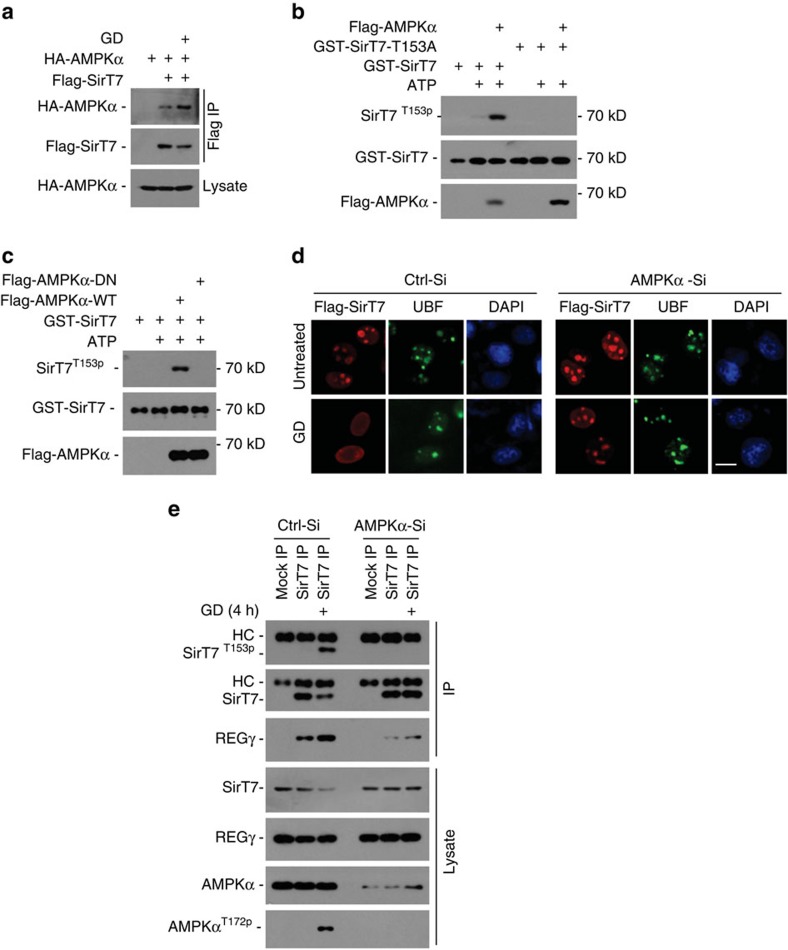
AMPK directly regulates SirT7 phosphorylation and subcellular distribution under starvation. (**a**) 293T cells transfected with Flag-SirT7 and HA-AMPKα plasmids were treated with or without GD (4 h), followed by immunoprecipitation with FLAG-M2 beads. The precipitated proteins were analysed by western blot using anti-Flag or anti-HA antibody. (**b**) *In vitro* phosphorylation of SirT7 by activated AMPKα. GST-SirT7-WT or -153A mutants were expressed in *E.coli* and purified with GST beads. Activated Flag-AMPKα was precipitated from GD (6 h) treated Flag-AMPKα-overexpressing 293T cells using FLAG-M2 beads and eluted with Flag peptide. GST-SirT7-WT or -153A proteins were incubated with or without Flag-AMPKα in the presence or absence of ATP as indicated. The reaction product was separated by SDS-PAGE and analysed by western blot. (**c**) Similar *in vitro* kinase assay was performed as detailed for (**b**), except that the kinase-dead AMPKα D159A (AMPK-DN) mutant was used. (**d**) HeLa cells with AMPKα knockdown (AMPKα-Si) or control Si-RNA (Ctrl-Si) were transfected with Flag-SirT7 following GD (12 h) treatment, then immunostained with anti-Flag (red) or anti-UBF (green) antibodies (scale bar, 10 μm). Nuclei were stained with DAPI. (**e**) HeLa cells with AMPKα knockdown or control Si-RNA were treated with GD (4 h) followed by immunoprecipitation with anti-SirT7 antibody and western blot analysis. AMPK activation was confirmed by its phosphorylation at Thr-172.

Finally, we investigated whether AMPK directly regulates SirT7 phosphorylation and subcellular distribution under starvation. Results showed that SirT7 coprecipitated with AMPKα in 293 T cells overexpressing Flag-SirT7 and HA-AMPKα under normal and GD conditions (Fig. 7a), the WT but not the kinase-dead (D159A) AMPKα phosphorylated SirT7-T153 *in vitro* (Fig. 7b,c), and AMPK knockdown (Si) markedly blocked GD-induced Flag-SirT7 nucleoplasmic redistribution (Fig. 7d) and GD-induced endogenous SirT7 phosphorylation and its association with REGγ (Fig. 7e). Collectively, these data indicate that AMPK-induced SirT7-T153 phosphorylation is crucial in stimulating REGγ-dependent SirT7 nucleoplasmic redistribution and degradation under energy starvation conditions.

### REGγ reduction sensitizes tumour to 2DG treatment *in vivo*

**Figure 8:**
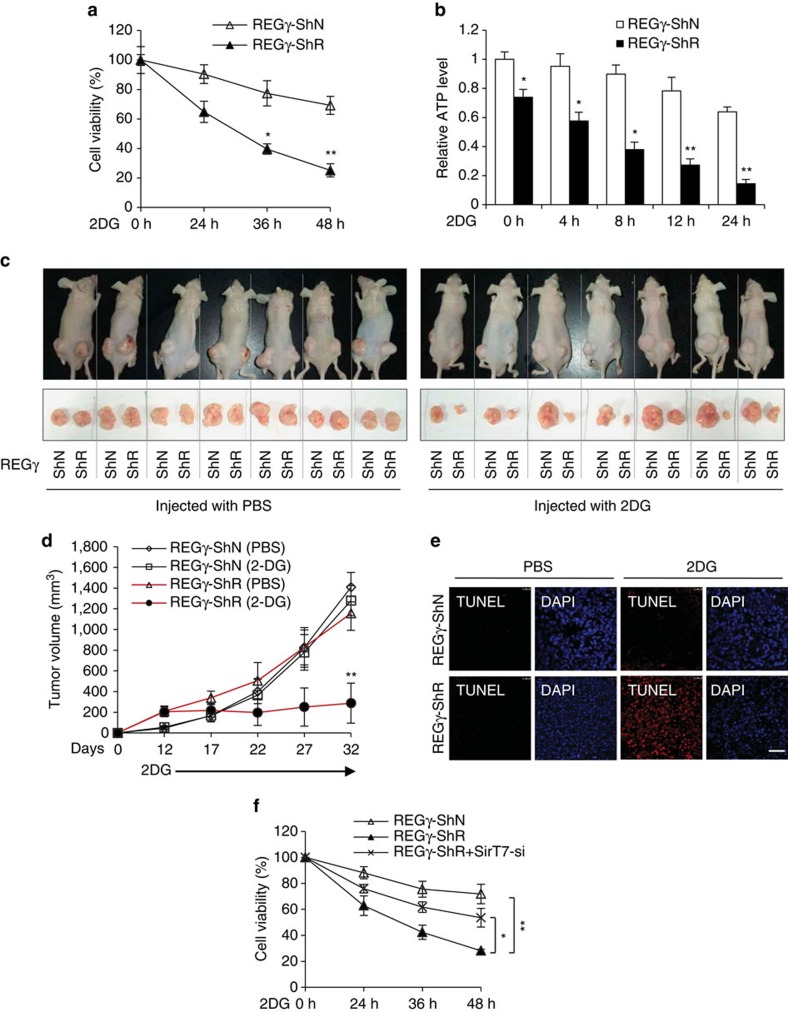
REGγ deficiency benefits 2DG treatment in tumour starvation. (**a**,**b**) REGγ knockdown enhances 2DG-induced cell death and ATP consumption. Stable REGγ-ShN and-ShR HCT116^−/−^ cells were treated with 2DG (12.5 mM) for the indicated time periods, and relative cell viability (**a**) and intracellular ATP levels (**b**) were determined. Data represent mean±s.d., *n*=3, *P<0.05, **P<0.01, Student's *t* test. (**c**-**e**) REGγ knockdown sensitizes the tumour to 2DG treatment in mice. (**c**,**d**) Mice with xenograft tumours originated from stable HCT116^−/−^ cells with REGγ-ShN or -ShR were treated with 2DG or PBS by intraperitoneal injection. Images show tumours after 32 days of PBS or 2DG treatment (**c**). The tumour size was measured every 5 days and tumour volume was calculated (**d**). Data represent mean±s.d., *n*=7, **P<0.01, Student's *t* test. (**e**) Tissue sections of xenograft tumours of mice on day 32 were analysed by TUNEL staining (Scale bar, 200 μm). Nuclei were stained with DAPI. (**f**) stable REGγ-ShN and-ShR HCT116^−/−^ cells infected with or without SirT7 knockdown (Si) lentivirus were treated with 2DG (12.5 mM) for the indicated times, and cell viability was determined using MTT assay. Data represent mean±s.d., *n*=3, *P<0.05, **P<0.01, Student's *t* test.

Targeting cancer cell metabolism is a promising strategy to fight cancer^38^. Previous studies indicated that 2DG treatment induces intracellular ATP depletion and thus sensitizes cancer cells to the treatment of radiation or chemotherapeutic agents^39^,^40^. Given our results strongly suggest that REGγ benefits energy saving and cell survival during energy stress, we tested whether 2DG is more effective in killing REGγ-knockdown cancer cells. Results showed that 2DG treatment led to a marked increase in cell death coupled with fast ATP consumption in REGγ knockdown HCT116^−/−^ (p53 null) cells (Fig. 8a,b), indicating that 2DG is more deleterious to REGγ-downregulated cells.

Next, we tested whether REGγ knockdown could enhance the therapeutic response of xenograft tumours in mice on 2DG treatment. REGγ-normal and -knockdown HCT116^−/−^ cells were injected into nude mice subcutaneously above the left and right hind legs separately. To avoid the potential growth retardation of REGγ-knockdown cells, more REGγ-knockdown cells were injected into the mice to develop tumours. Results showed that the tumour growth was markedly inhibited in REGγ-knockdown groups receiving 2DG but not PBS treatment (Fig. 8c,d). TUNEL staining of tumour sections showed that 2DG treatment markedly increased cell death in REGγ-knockdown cancer cells (Fig. 8e). These results indicate that knockdown of REGγ sensitizes tumour to energy starvation. In addition, we observed that double knockdown of SirT7 significantly reduced 2DG-induced cell death in stable REGγ-knockdown HCT116^−/−^ cells (Fig. 8f), suggesting that endogenous SirT7, at least in part, contributes to energy stress induced tumour cell death in REGγ-knockdown cells.

## Discussion

Our findings reveal a previously unknown function of the REGγ proteasome in the control of energy homeostasis. REGγ deficiency results in an increase in energy consumption via increasing rDNA transcription. Moreover, energy starvation can activate an AMPK-dependent SirT7 phosphorylation, which leads to a REGγ-dependent nucleoplasmic retaining and degradation of SirT7, and thereby switching off rDNA transcription to save energy to maintain cell survival. Furthermore, REGγ deficiency significantly improves the anti-tumour activity of 2DG *in vivo*. These findings present the first evidence that the Ub-independent proteasome pathway is directly involved in the regulation of rDNA transcription and energy balance, suggesting that inhibition of the REGγ proteasome has the potential to enhance the tumour-killing efficacy of energy starvation.

Previous studies identified that both the Ub and the 19S ATPase subunits of the proteasome were observed within the nucleoli^41^; however, no 26S proteasome or the 20S catalytic core can be detected in the nucleoli^5^,^41^. Our results show that REGγ interacts with SirT7, promotes its Ub-independent degradation and thereby limits rDNA transcription under normal conditions. Furthermore, upon starvation, AMPK can increase the phosphorylation state of SirT7 dramatically, leading to REGγ-dependent SirT7 nucleoplasmic redistribution and degradation, and thus facilitating rDNA transcription silencing. These observations provide a mechanism of how the proteasome system is positively involved in limiting and turning off rDNA transcription under normal and energy starvation conditions. Since inhibition of rDNA transcription represents the major ribosome biogenesis-limiting and energy-saving strategy to maintain cellular homeostasis under stress conditions^42^,^43^, we conclude that REGγ proteasome not only regulates protein turnover in an energy-saving manner, but also prevents excessive protein synthesis and energy waste via limiting rDNA transcription, and thus provides a simple protein and energy homeostatic control mechanism for the Ub-independent proteasome system.

A recent report suggests that inhibition of pre-rRNA synthesis leads to the release of SirT7 from nucleoli^37^. However, whether SirT7 nucleolar delocalization is the cause or the consequence of rDNA transcription silencing remain unclear. In this study, we reveal that (i) REGγ overexpression causes a marked nucleoplasmic relocalization of SirT7, (ii) REGγ deficiency significantly blocks GD- and AICAR-induced nucleoplasmic redistribution of SirT7, (iii) ActD treatment increases REGγ-SirT7 association and REGγ-dependent SirT7 degradation, (iv) elimination of SirT7 phosphorylation at T153 blocks GD-induced nucleoplasmic relocalization of SirT7, and (v) both SirT7-T153D/E mutant and starvation-induced T153 phosphorylated SirT7 exhibit predominant nucleoplasmic localization in the REGγ-normal cells, but predominant nucleolar localization in the REGγ knockdown cells. These observations clearly demonstrate that catching nucleoplasmic phosphorylated SirT7 by REGγ serves as a major mechanism to control SirT7 redistribution and degradation under energy stress conditions. However, our study does not exclude that the shortage of nascent pre-rRNA under energy stress conditions may further facilitate nucleoplasmic REGγ-SirT7 association and SirT7 degradation. Notably, we also reveal that SirT7 can be modified by ubiquitination or by phosphorylation at other sites including S54, S166 and T284. It will be interesting to further study whether these modifications may specifically affect the turnover and function of SirT7 under other stress conditions.

Intriguingly, previous studies of Sirtuins in rDNA transcription revealed that SirT7 serves as the activator in the regulation of rDNA transcription^25^,^26^ whereas SirT1 represses rDNA transcription during caloric restriction^4^. Our previous study revealed that REGγ also binds to and promotes degradation of SirT1 (ref. 12). In this study, we observed that knockdown of SirT7 markedly blocked REGγ deficiency-induced upregulation of rDNA transcription, indicating that SirT7 plays a predominant role in determining the levels of rDNA transcription in the REGγ-deficient cells under normal conditions. Supportingly, another previous study proved that treatment of Sirtinol (inhibitor of all sirtuins, including SirT1 and SirT7) caused a dramatic decrease in nucleolar transcription, and SirT7 knockdown significantly decreased rDNA transcription whereas SirT1 knockdown led to no clearly increase of rDNA transcription under normal cell culture conditions^26^. In this study, we also reveal that energy starvation induces AMPK-dependent SirT7 T153 phosphorylation, which further promotes REGγ-dependent SirT7 nucleoplasmic redistribution and degradation, leading to silencing of rDNA transcription. Of note, our previous study revealed that glucose starvation also induces AMPK-dependent SirT1 phosphorylation at T530, which entails SirT1-REGγ dissociation^12^. Since SirT1 was reported to repress rDNA transcription during caloric restriction^4^, we also suggest that starvation-induced SirT1 dissociation from REGγ also contributes to rDNA transcription inhibition upon starvation. Since the AMPK-induced phosphorylation of SirT1/SirT7 leads to diverse REGγ-SirT1/SirT7 binding, we conclude that REGγ acts as a central hub in coordinating AMPK signalling pathway to effectively turning off rDNA transcription to save energy required for the maintenance of cell survival under conditions of energy shortage.

Our study also reveals that the REGγ-proteasome is strongly linked to tumour-starving cancer therapy. Caloric restriction has long been considered as a promising approach for cancer therapy. However, in response to starvation, cells always reduce energy consuming, and thereby decrease the effects of nutrient limitation on killing tumours. For example, the eNoSC-induced epigenetic rDNA silencing contributes to protection of cells from glucose starvation-induced apoptosis^4^. Thereby, the intracellular energy-saving response impairs the effectiveness of metabolism inhibitors in killing tumours. In this study, we show that knockdown of REGγ is sufficient to promote both GD- and 2DG-induced energy consumption and cell death, and, as a result, increases tumour killing efficacy of 2DG *in vivo*. REGγ has been shown to protect cells against apoptosis by inhibiting p53 (ref. 31), but our results here show that p53 is not essential for starvation-induced cell death in REGγ-deficient cells. In contrast, we observed that double knockdown of SirT7 significantly decreased GD- and 2DG-induced cell death in REGγ-knockdown cells, indicating that the endogenous SirT7, at least in part, contributes to starvation-induced cell death in REGγ-deficient cells. However, there remains the possibility that REGγ protects cells from starvation-induced cell death via other pathways as well. Of note, aberrant expression of REGγ was observed in some types of tumours^44^; thus it is tempting to further investigate whether pharmacological manipulation of REGγ could provide cancer-starving benefits.

In summary, we identify a role of REGγ proteasome in rDNA transcription, energy consumption and cell fate decision under energy stress conditions. This study expands the current knowledge of Ub-independent proteasome in protein and energy homeostasis. In addition, knockdown of REGγ significantly increased 2DG-induced tumour growth inhibition and cancer cell death both *in vivo* and *in vitro*. These results lead us to propose that specific inhibition of REGγ proteasome may restore tumour sensitivity to energy starvation.

## Methods

### Cell culture and materials

HeLa, HCT116, HCT116-p53 null (HCT116^−/−^) and HEK293 T (293T) cells were obtained from the American Type Culture Collection. REGγ-WT and REGγ-KO MEF cells were derived from C57BL/6 WT and REGγ knockout (KO) mice. All cells were maintained in DMEM with 10% (v/v) fetal bovine serum. REGγ-knockdown plasmids were previously generated^12^. The small hairpin RNA (ShRNA) was constructed using the following primers: REGγ-ShR1 (also written as ShR)-F: 5′- CCGGGTGAGGCAGAAGACTTGGTGGCTCGAGCCACCAAGTCTTCTGCCTCACTTTTTG -3′. REGγ-ShR1-R: 5′- AATTCAAAAAGTGAGGCAGAAGACTTGGTGGCTCGAGCCACCAAGTCTTCTGCCTCAC -3′. REGγ-ShR2-F: 5′- CCGGGAGTCTGGCTCAAGACCGACACTCGAGTGTCGGT CTTGAGCCAGACTCTTTTTG -3′. REGγ-ShR2-R: 5′- AATTCAAAAAGAGTCTGGCTCAAGACCGACACTCGAGTGTCGGTCTTGAGCCAGACTC -3′. SirT7-Si-F: 5′- CCGGCTTCAGAAAGGGAGAAGCGTTCTCGAGAACGCTTCTCCCTTTCTGAAGTTTTTG -3′. SirT7-Si-R: 5′- AATTCAAAAACTTCAGAAAGGGAGAAGCGTTCTCGAGAACGCTTCTCCCTTTC TGAAG -3′. REGγ deletion mutants were constructed by PCR from GFP-REGγ. HA-AMPKα1 and Flag-tagged UBF, AMPKα1 and MYBBP1A expression plasmids were constructed by inserting the coding regions into pCDNA3.0 vector, respectively. For SirT7 expression plasmid construction, a short form of SirT7 (missing residues 33–78) was amplified from human cDNA and inserted to pCDNA3.0-Flag, pCDNA3.0-GFP and PGEX4T-1 vectors. To generate the canonical form of SirT7, the coding sequences of residues 33–78 of SirT7 were amplified from human genomic DNA and inserted into the plasmid-expressing short form of SirT7. SirT7 and AMPKα1 (D159A) mutants were generated using site-directed mutagenesis. 3MA, 2DG, AICAR, methyl pyruvate, Flag M2 beads, Compound C and APDC were obtained from Sigma. Antibodies were purchased from Sigma (Flag, F3165, 1/5,000 dilution; β-actin, A5441, 1/5,000 dilution), Santa Cruz (GFP, sc-8334, 1/3,000 dilution; SirT7, sc-135055, 1/1,000 dilution), Proteintech (GST, 10000-0-AP, 1/5,000 dilution; His, 10001-0-AP, 1/2,000 dilution), Abcam (SirT7, ab62748, 1/1,000 dilution), BD (REGγ, 611181, 1/2,000 dilution), CST (P-Thr/Ser, 9631S, 1/500 dilution; p-AMPK^T172^, 2535 T, 1/500 dilution) and Bioword (AMPK, BS1009, 1/1,000 dilution; Caspase 3, BS7004, 1/500 dilution). A rabbit antiserum against SirT7 phosphorylated at T153 was raised against peptide MSIT (P) RLHEQKLC, of which the threonine is phosphorylated and indicated as T (P). The antiserum was pre-cleaned using the corresponding non-phosphorylated peptide coupled to Sulfolink coupling resin (Thermo Scientific) and purified by affinity chromatography. Uncropped images of the original scans of representative immunoblots are shown in Supplementary Fig. 5.

### Immunoprecipitation and western blotting

Cell lysate preparation and western blotting were performed as previously described^45^. Briefly, cells were lysed in lysis buffer (50 mM Tris-HCl pH 8.0, 5 mM EDTA, 150 mM NaCl, 0.5% NP-40, 1 mM PMSF), centrifuged for 5 min at 10,000 g, and the insoluble debris was discarded. Cell lysates were further analysed with SDS-PAGE and western blotting. For co-immunoprecipitation, cell lysates were immunoprecipitated with anti-Flag-M2 agrose or Protein A/G agrose plus anti-REGγ or anti-SirT7 antibodies for 4–6 h at 4 °C. The beads were washed extensively with lysis buffer, boiled in SDS sample buffer, fractionated by SDS-PAGE, and analysed by western blotting using specific antibodies.

### Immunofluorescence staining

Immunofluorescence was performed as described in detail previously^46^. Briefly, cells were fixed in 4% formaldehyde, immunostained for 2 h with primary antibodies followed by a 2 h exposure to Alexa Fluor 488- or 568-conjugated secondary antibodies. Immunofluorescence was visualized by fluorescence microscopy.

### Measurement of ATP level and ADP/ATP ratio

ATP levels were measured using ATP assay kit (Beyotime). The ADP/ATP ratio was analysed using the ADP/ATP assay kit (Sigma).

### *In vivo* ubiquitination assay

293 T cells seeded on 100 mm dishes were transfected as indicated. The ubiquitinated proteins were purified as previously described^45^. Briefly, cells in 10 cm plates were co-transfected with plasmids encoding His6-ubiquitin, Flag-SirT7 or GFP-REGγ expression plasmids as indicated for 24 h and treated with or without 25 μM MG132 for 4 h before harvest. Cells were lysed in buffer A (6 M guanidinium-HCl, 0.1 M Na_2_HPO_4_/NaH_2_PO_4_, 0.01 M Tris-HCl pH 8.0, 5 mM imidazole, 10 mM β-mercaptoethanol) and incubated with Ni-NTA beads (Qiagen) for 4 h at room temperature. The beads were washed with buffer A, B (8 M urea, 0.1 M Na_2_PO_4_/NaH_2_PO_4_, 0.01 M Tris-HCl pH 8.0, 10 mM β-mercaptoethanol), C (8 M urea, 0.1 M Na_2_PO_4_/NaH_2_PO_4_, 0.01 M Tris-HCl pH 6.3, 10 mM β-mercaptoethanol), and bound proteins were eluted with buffer D (200 mM imidazole, 0.15 M Tris-HCl pH 6.7, 30% glycerol, 0.72 M β-mercaptoethanol, 5% SDS). The eluted proteins were analysed by western blot for the presence of conjugated Flag-SirT7 by anti-Flag antibody. To confirm the expression of transfected plasmids, 20% of the cells were harvested and were lysed in RIPA buffer for western blotting.

### Luciferase reporter assay

To generate rDNA promoter-activated luciferase reporter, −508 to +200 bp of rDNA promoter (the transcription start site is represented as +1) was subcloned into PGL4.17. Luciferase reporter assay was performed as previously described^46^. Briefly, cells in 24-well plates were cotransfected with indicated plasmids and the rDNA-promoter luciferase reporter plasmid for 24 h. Luciferase was measured using the Dual-Luciferase assay kit (Promega). pRL-TK was co-transfected to normalize transfection efficiency.

### Cell death analyses

Cell survival was determined by MTT assay. Apoptosis analyses were measured by annexin V FITC/PI assay using the flow cytometry as previously described^46^. Briefly, cells were stained using the Annexin V-FITC/propidium iodide apoptosis detection kit and measured using a BD Biosciences FACS Aria flow cytometer. Necrosis-associated DNA degradation was performed as previously described^47^. Briefly, cells were lysed with lysis buffer (20 mM EDTA, 100 mM Tris-HCl pH 8.0, 0.8% SDS) followed by RNase A (60 μg ml^−1^, 37 °C, 1 h) and Proteinase K (120 μg ml^−1^, 50 °C, 5 h) treatment. The samples were electrophoresed for 2 h at 60 V in 0.5% agarose gel.

### Pre-rRNA analysis

Pre-rRNA (47S/45S) levels were measured by qRT-PCR analysis and normalized against the expression levels of β-actin (internal control) using the following PCR primers: pre-rRNA (5′- GAACGGTGGTGTGTCGTTC -3′ and 5′- GCGTCTCGTCTCGTCTCA CT -3′); β-actin (5′- TCCTGTGGCATCCACGAA -3′ and 5′- TCGTCATACTCCTGCTTGC -3′). The levels of pre-rRNA and β-actin were also determined by northern blots using specific biotin-conjugated oligonucleotide probes and HRP-conjugated streptavidin. The filters were developed using ECL-plus reagent. The biotin-conjugated riboprobes were: human pre-rRNA (5′-biotin- CGAACCCCACACCGACGAGCTCC -3′); human β-actin (5′-biotin- TAGGATGGCAAGGGACTTCCTG -3′); mouse pre-rRNA (5′-biotin- AGAGAAAAGAGCGGAGGTTCGGGACTCCAA -3′) and mouse β-actin (5′-biotin- GGGTGCTCCTCAGGGGCCACA -3′).

### *In vitro* phosphorylation assay

*In vitro* phosphorylation assay was performed as previously described^46^. Briefly, 293 T cells overexpressing Flag-tagged WT AMPKα1 (WT) or AMPKα1 kinase-dead mutant (D159A) were treated with glucose-free DMEM for 6 h before harvest. Then AMPKα1 was precipitated from cell lysates using Flag-M2 beads and eluted with Flag-peptide (Sigma). GST-SirT7 (WT or T153A mutant) proteins were expressed in *Escherichia coli* BL21 (DE3) and purified using glutathione-Sepharose 4B resin (GE Healthcare). GST-SirT7 and Flag-AMPK (WT or D159A) complex in kinase buffer (25 mM Tris-HCl pH 7.5, 5 mM glycerophosphate, 2 mM DTT, 0.1 mM Na_3_VO_4_, 2 mM ATP and 10 mM MgCl_2_) were incubated at 30 °C for 30 min. The reaction was stopped by adding 2 × SDS-PAGE sample buffer and analysed by western blot.

### Tumour growth analysis in mice

REGγ-ShN (3 × 10^6^ cells) and REGγ-ShR (4.5 × 10^6^ cells) HCT116 p53 null (HCT116^−/−^) cells were subcutaneously injected into both flanks of 14 male BALB/c nude mice (∼5 weeks of age). Twelve days after injection, mice (7 per group) were treated with PBS or 2DG (700 mg kg^−1^) every day by intraperitoneal injection. Tumour size was measured every 5 days using calipers. At day 32, tumours were extracted from nude mice and measured by TUNEL assays using in situ Cell Death TMR (Roche) and visualized by microscopy. Animals were treated according to high ethical and scientific standards with oversight by the animal centre at East China Normal University.

### Mass spectrometry analysis

293 T cells maintained in normal growth medium were transfected with Flag-SirT7 plasmid. Transfected cells were harvested 24 h post-transfection and lysed. Flag-SirT7 was immunoprecipitated with Flag-M2 beads, eluted with Flag-peptide (Sigma), then subjected to SDS-PAGE and visualized by Coomassie blue staining. The Flag-SirT7 band was excised, distained and digested in 50 mM ammonium bicarbonate with 12.5 ng μl^−1^ trypsin. Peptide mixtures were analysed online with a hybrid Q-Exactive mass spectrometer. Mass spectra were searched using the SEQUEST algorithm against a Uniprot human database. All peptide matches were initially filtered based on enzyme specificity, mass measurement error, Xcorr and Corr scores and further manually validated for peptide identification and phosphorylation site localization.

### Statistical analyses

The results are expressed as mean±s.d. as indicated in the figure legends. Statistical significance was assessed by two-tailed Student *t* tests. Values of *P*<0.05 were considered significant.

### Data availability

The authors declare that the data supporting the findings of this study are available within the article and its Supplementary Information Files. All other relevant data supporting the findings of this study are available on request.

## Additional information

**How to cite this article:** Sun, L. *et al*. Regulation of energy homeostasis by the ubiquitin-independent REGγ proteasome. *Nat. Commun.* 7:12497 doi: 10.1038/ncomms12497 (2016).

## Supplementary Material

Supplementary Information Supplementary Figures 1-5
