# Supplementary material for: Regulation of energy homeostasis by the ubiquitin-independent REGγ proteasome
Source: Nat Commun. 2016 Aug 11;7:12497. doi: 10.1038/ncomms12497 (PMC4987533; doi:10.1038/ncomms12497)
Supplement: Supplementary Information — Supplementary Figures 1-5 [file ncomms12497-s1.pdf]

Supplementary Figure 1

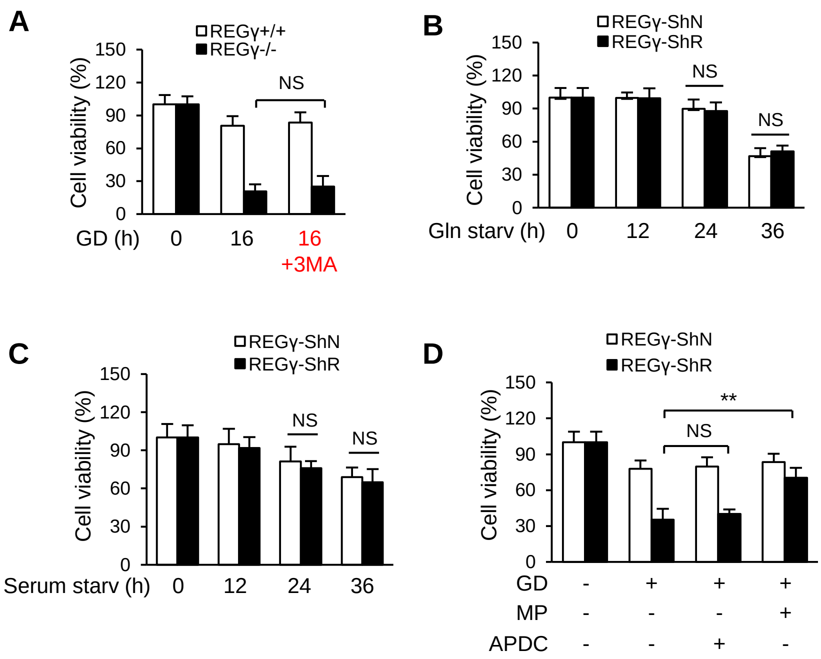

Supplementary Figure 1. Effects of different stresses on the cell death of REGγ deficient cells. (A) REGγ-WT and -KO MEF cells were treated with glucose deprivation (GD, 16h) in the presence or absence of 3MA (20 μM). Cell viability was detected by MTT assay. (B, C) HeLa cells with stable knockdown of REGγ (ShR) or a vector control (ShN) were cultured in glutamine-free (Gln starv) (B) or serum free (Serum starv) (C) DMEM-high glucose medium for indicated times, and the cell viability was examined using MTT assay. (D) Cells in (B) were pretreatment with or without ROS inhibitor APDC (50 μM, 30 min) followed by glucose deprivation (GD, 24h) in the presence or absence of methyl pyruvate (MP), and the cell viability was examined using MTT assay. All experiments were repeated three times, data represent mean ±s.d., **P < 0.01, NS= not significant, Students t-test.

Supplementary Figure 2

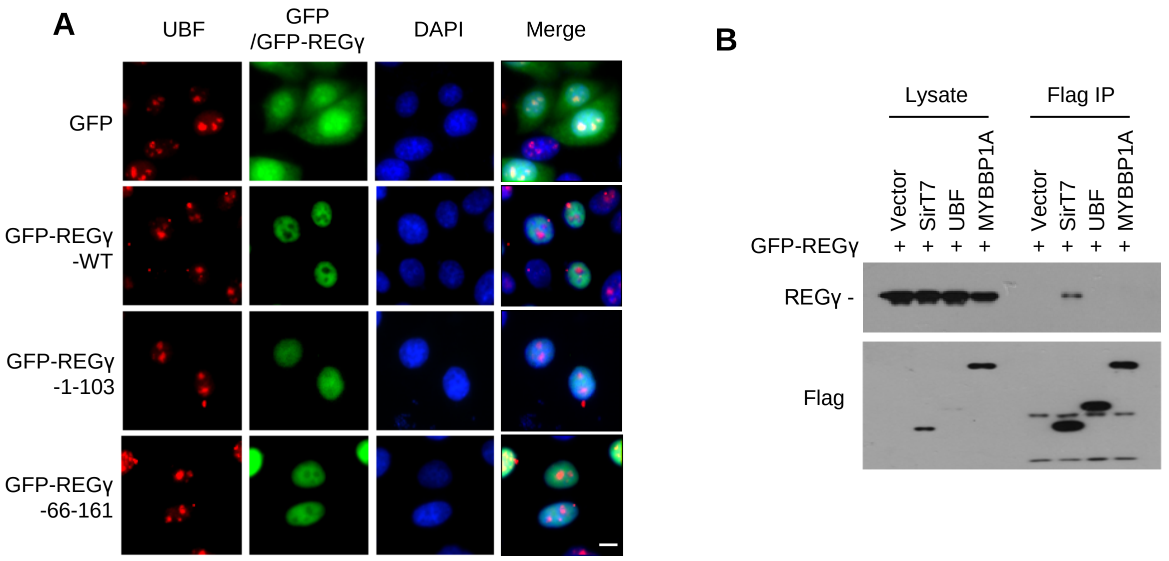

Supplementary Figure 2. The specificity of REGγ-SirT7 interaction. (A) GFP-REGγ plasmids (wildtype, aa1-103, or aa66-161) were transfected to HeLa cells, and the endogenous UBF was immunostained with anti-UBF antibody (red) and visualized by fluorescence microscopy (scale bar, 10 µm). GFP-REGγ was detected by the intrinsic fluorescence of GFP. Nuclei were stained with DAPI. (B) Flag-tagged rDNA transcription regulators (SirT7, UBF and MYBBP1A) and GFP-REGγ were cotransfected into 293T cells and immunoprecipitated with FLAG-M2 agarose beads, the coprecipitated REGγ was detected by western blot using anti-GFP antibody.

Supplementary Figure 3

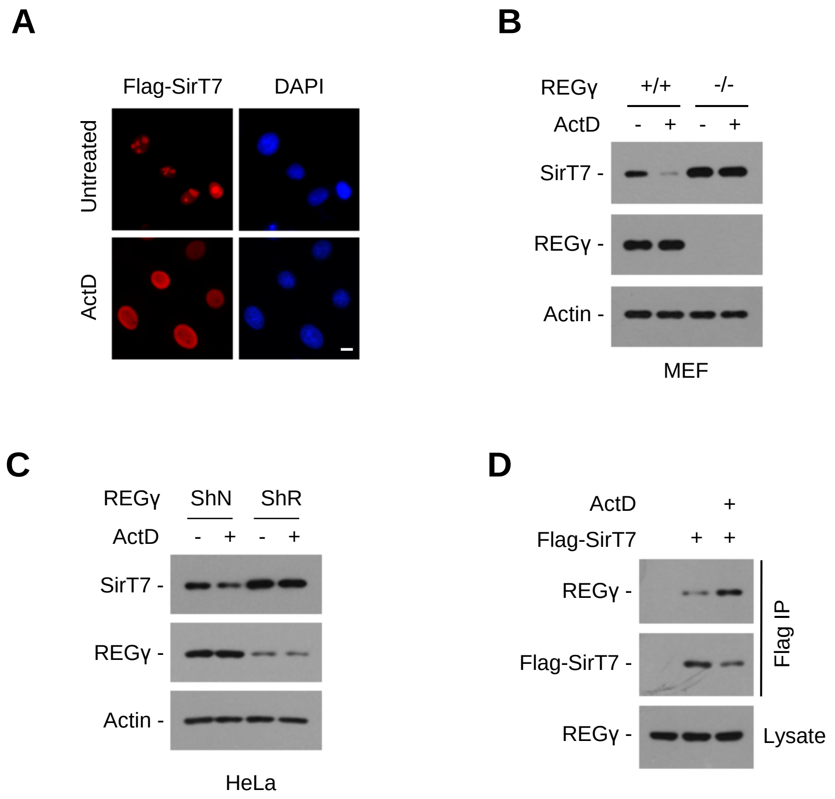

Supplementary Figure 3. REGγ regulates SirT7 degradation upon ribosomal stress. (A) Ribosomal stress causes nucleolar delocalization of SirT7. HeLa cells transfected with Flag-SirT7 and GFP were treated with actinomycin D (ActD, 50ng ml^{-1}, 2h) and immunostained with Flag antibody (red). Nuclei were stained with DAPI in blue (scale bar, 10 μm). (B, C) REGγ promotes SirT7 degradation under ribosomal stress. REGγ-WT and -KO MEF cells (B) or REGγ-ShN and -ShR HeLa cells (C) were treated with ActD (50ng ml^{-1}, 2h) and analyzed for SirT7 expression by western blot. (D) Ribosomal stress increases REGγ-SirT7 association. 293T cells overexpressing Flag-SirT7 were treated with ActD (50ng ml^{-1}, 2h). REGγ-SirT7 complex was detected by immunoprecipitation using FLAG M2 beads followed by anti-REGγ western blot.

Supplementary Figure 4

A

Site	peptide
S54	LLAES(p)ADLVTELQGR
S166	LVQHVVS(p)QNCDGLHLR
T284	LWCMT(p)KPPSR

B

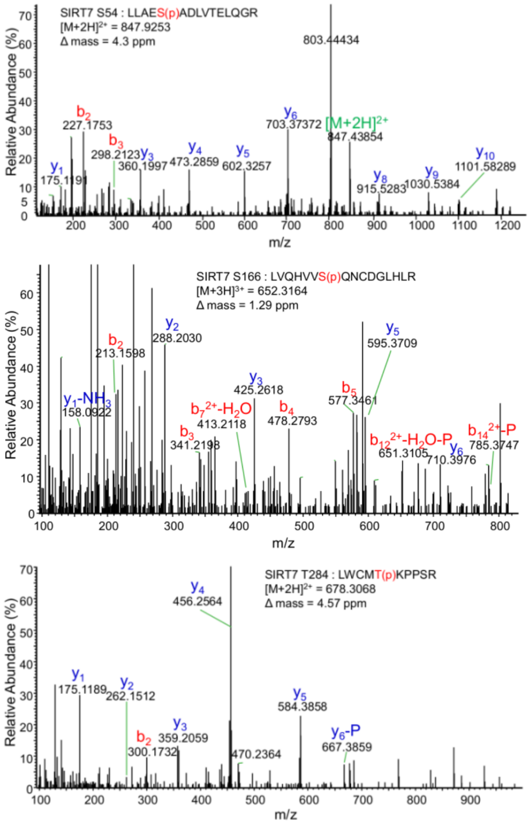

C

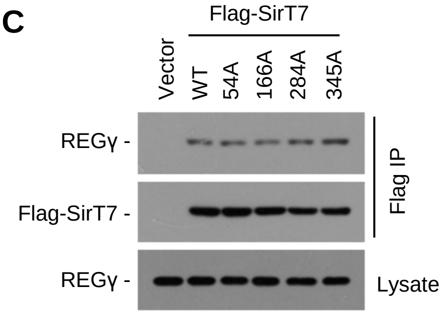

D

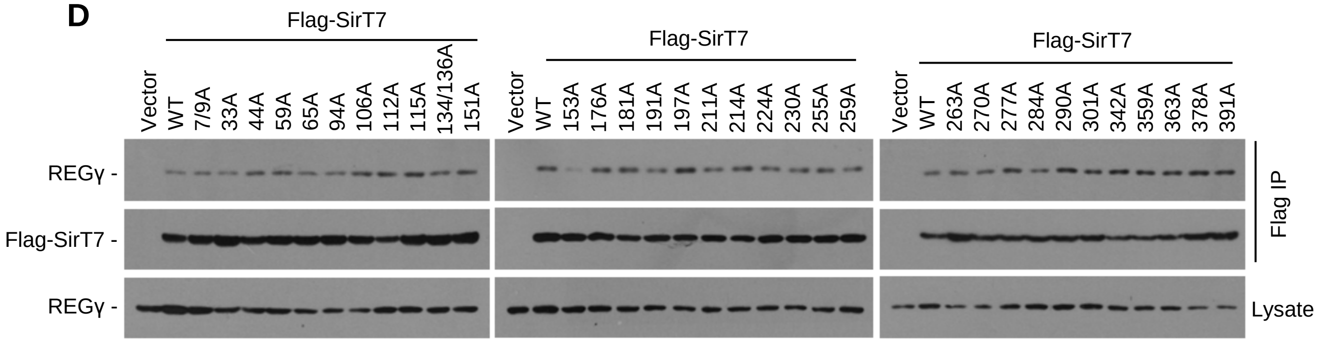

E

T153

Homo	HMSI**T**RLHEQKLVQH
Mus mouse	HMSI**T**RLHEQKLVQH
Norway rat	HMSI**T**QLHEQKLVQH
Rhesus monkey	HMSI**T**RLHEQKLVQH
Olive baboon	HMSI**T**RLHEQKLVQH
Cattle	HMSI**T**RLHEQKLVQH

F

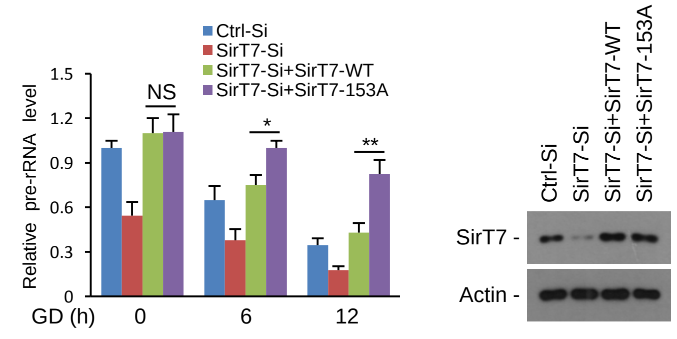

Supplementary Figure 4. Identification of potential phosphorylation sites in SirT7 regulating REGγ-SirT7 binding. (A) Potential phosphorylation sites in SirT7 as revealed by mass spectrometry analysis. (B) The mass spectra of the SirT7 peptides phosphorylated at Ser54, Ser166 and Thr284. (C, D) Flag-SirT7-wildtype (WT) or indicated Flag-SirT7 mutant (S/T to A) plasmids were transfected to 293T cells and immunoprecipitated with FLAG-M2 agarose beads, the coprecipitated REGγ was detected by western blot using anti- REGγ antibody. (E) SirT7 Thr153 is highly conserved in mammals. (F) SirT7-T153A recomplementation attenuates starvation-induced reduction of pre-rRNA. HCT116$^{-/-}$ cells with si-control (Ctrl-Si), SirT7 knockdown (SirT7-Si) or SirT7-Si restored with lentiviral vectors expressing SirT7-WT or SirT7-153A were treated with glucose deprivation (GD) for 6-12h. The relative pre-rRNA levels were analyzed by qRT-PCR. Western blots showing SirT7 expression. All experiments repeated three times, data represent mean ± s.d., NS=non-significant, *$P < 0.05$, **$P < 0.01$, Students t-test.

Supplementary Figure 5

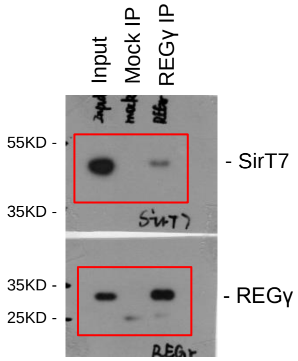

Figure 3D

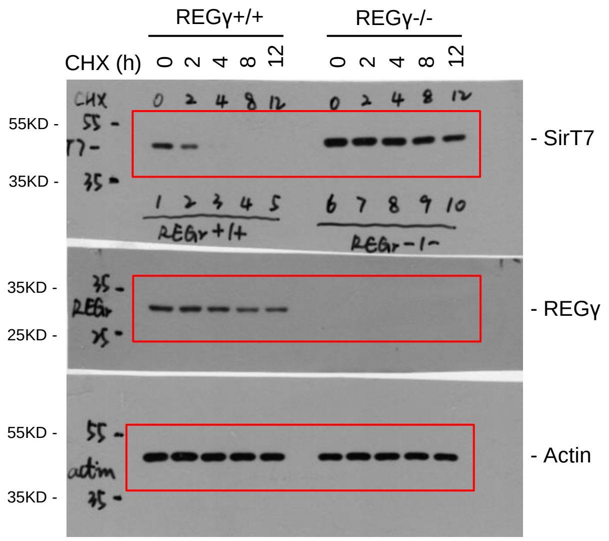

Figure 3I

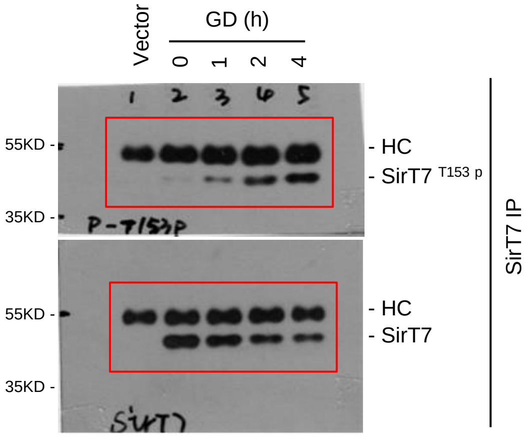

Figure 6F

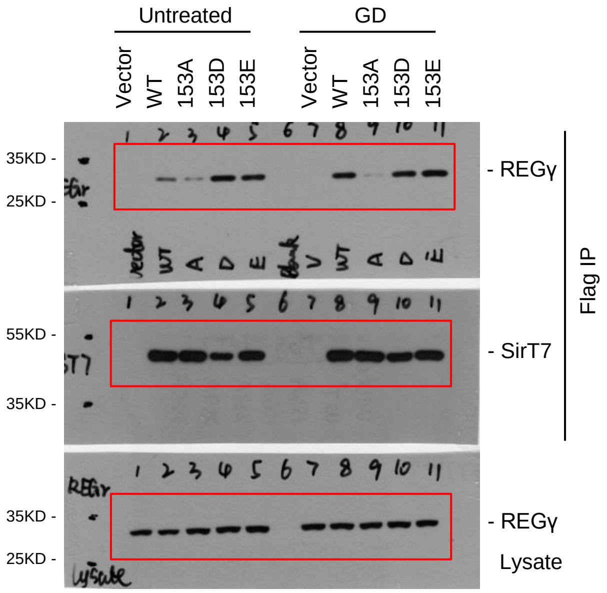

Figure 6J

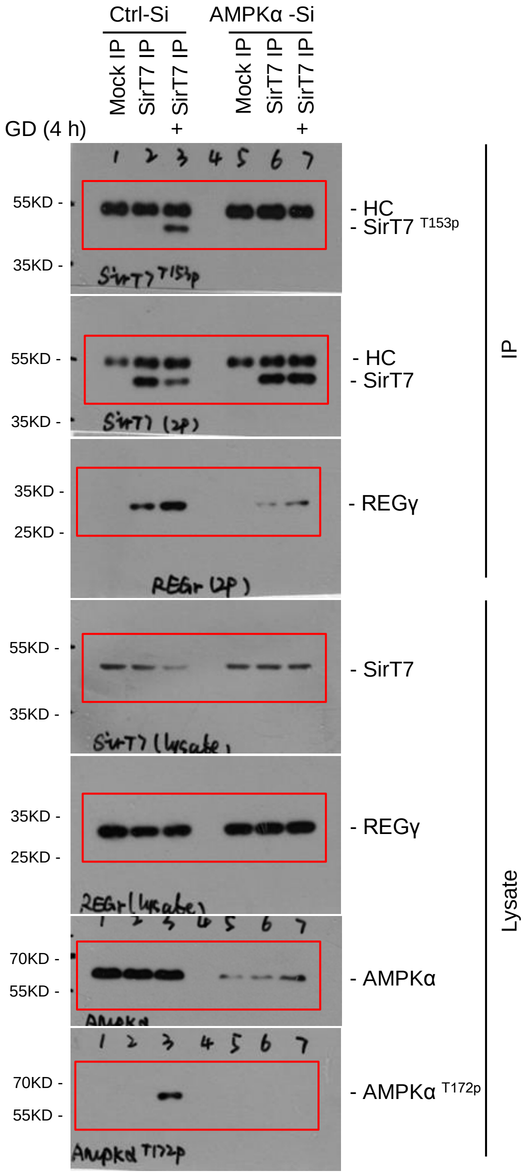

Figure 7E

Supplementary Figure 5. Uncropped images of the original scans of representative immunoblots. Red boxes indicate the cropped areas displayed in the indicated figures.
